# MnPSe_3_ Monolayer: A Promising 2D Visible‐Light Photohydrolytic Catalyst with High Carrier Mobility

**DOI:** 10.1002/advs.201600062

**Published:** 2016-04-23

**Authors:** Xu Zhang, Xudong Zhao, Dihua Wu, Yu Jing, Zhen Zhou

**Affiliations:** ^1^ Tianjin Key Laboratory of Metal and Molecule Based Material ChemistryComputational Centre for Molecular ScienceInstitute of New Energy Material ChemistryCollaborative Innovation Center of ChemicalScience and Engineering (Tianjin)School of Materials Science and EngineeringNational Institute for Advanced MaterialsNankai UniversityTianjin300350P. R. China

**Keywords:** 2D materials, carrier mobility, MnPSe_3_, visible light, water splitting

## Abstract

**The 2D material single‐layer MnPSe_3_** would be a promising photocatalyst for water splitting, as indicated by the proper positions of band edges, strong absorption in visible‐light spectrum, broad applicability (pH = 0 – 7), and high carrier mobility.

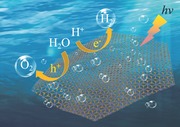

Since the experimental realization of graphene,^1^ 2D materials have been receiving significant attention due to their unique physical and chemical properties which mainly arise from their topological effects and high surface‐bulk ratios. Graphene has predominated as the most widely studied 2D material. Intractably, lack of a band gap limits its practical applications to high speed switching devices, photocatalysts, etc.^2^ Nevertheless, the successful preparation of graphene has prompted researchers to investigate more 2D materials such as hexagonal BN,^3^ transition metal dichalcogenides (TMDs),^4^ silicene,^5^ germanane,[[qv: 5b]],^6^ phosphorene,^7^ and MXene.^8^ These 2D materials attract intensive interest due to their novel electronic, mechanical or photocatalytic behaviors,^9^ making up the shortages of graphene and expanding the applications of 2D materials to field‐effect transistors (FETs)^10^ and photocatalysts.^11^

Recently, a new kind of 2D materials (exemplified by MnPSe_3_) was proposed by Li et al.^12^ Then, a series of MPS_3_ (M = Fe, Mn, Ni, Cd, Zn) and MPSe_3_ (M = Fe, Mn) were explored by Du et al.^13^ Particularly, bulk crystals and few‐layer samples of MPX_3_ (X = S, Se) were obtained and characterized in experiments. The previous reports indicated that the band gaps of these MPX_3_ bulks range from 1.3 to 3.5 eV,^13^ suggesting the light absorption at a wide wavelength for photocatalysts.

Hydrogen generation by photocatalytic water splitting would present a promising method for solar energy conversion and play a very important role in solving serious environmental problems.^14^ However, inability to utilize visible light, low quantum yield, and fast backward reaction limit the practical application of photocatalytic water splitting.^15^ 2D materials with an appropriate band gap (i.e., ≈2–3 eV) would exhibit more efficient use of visible light in the photocatalytic process. Moreover, 2D nature means short distance for the generated electrons and holes to migrate, reducing the possibility of electron–hole recombination, and then giving high quantum yields. Enlightened by some 2D materials which are predicted to be photocatalysts for water splitting under visible light,[[qv: 15b]],^16^ and especially some proved good photocatalysts in experiments such as g‐C_3_N_4_,^17^ we investigated the band edges and optical properties of MPS_3_ (M = Fe, Mn, Ni, Cd, Zn) and MPSe_3_ (M = Fe, Mn) monolayers to screen more proper photocatalysts for water splitting. Then the carrier mobility was calculated through deformation potential (DP) theory for MnPSe_3_ monolayer which is a direct‐band‐gap semiconductor and has strong absorption in the visible‐light region. The high carrier mobility of MnPSe_3_ monolayer (up to 625.9 cm^2^ V^−1^ S^−1^) could be comparable to or even higher than those of many other 2D materials, indicating that the transfer of carriers to reactive sites would be easier in the photocatalytic process. Our results disclose that MnPSe_3_ monolayer would be a promising photocatalyst for water splitting under visible light.

The structural properties of MPS_3_ (M = Fe, Mn, Ni, Cd, Zn) and MPSe_3_ (M = Fe, Mn) monolayers were explored first. To determine the ground state of MPS_3_ (M = Fe, Mn, Ni, Cd, Zn) and MPSe_3_ (M = Fe, Mn), both spin‐unpolarized and spin‐­polarized computations were performed. The results show that spin‐polarized total energies are less favorable than spin‐­unpolarized ones for FePS_3_, CdPS_3_, ZnPS_3_, and FePSe_3_ mono­layers, indicating that these monolayers have nonmagnetic ground states. However, MnPS_3_, NiPS_3_, and MnPSe_3_ mono­layers prefer antiferromagnetic (AFM) coupling, which is more stable than the ferromagnetic (FM) state. The structures of these kinds of monolayers are similar, as shown in **Figure** **1**a.

**Figure 1:**
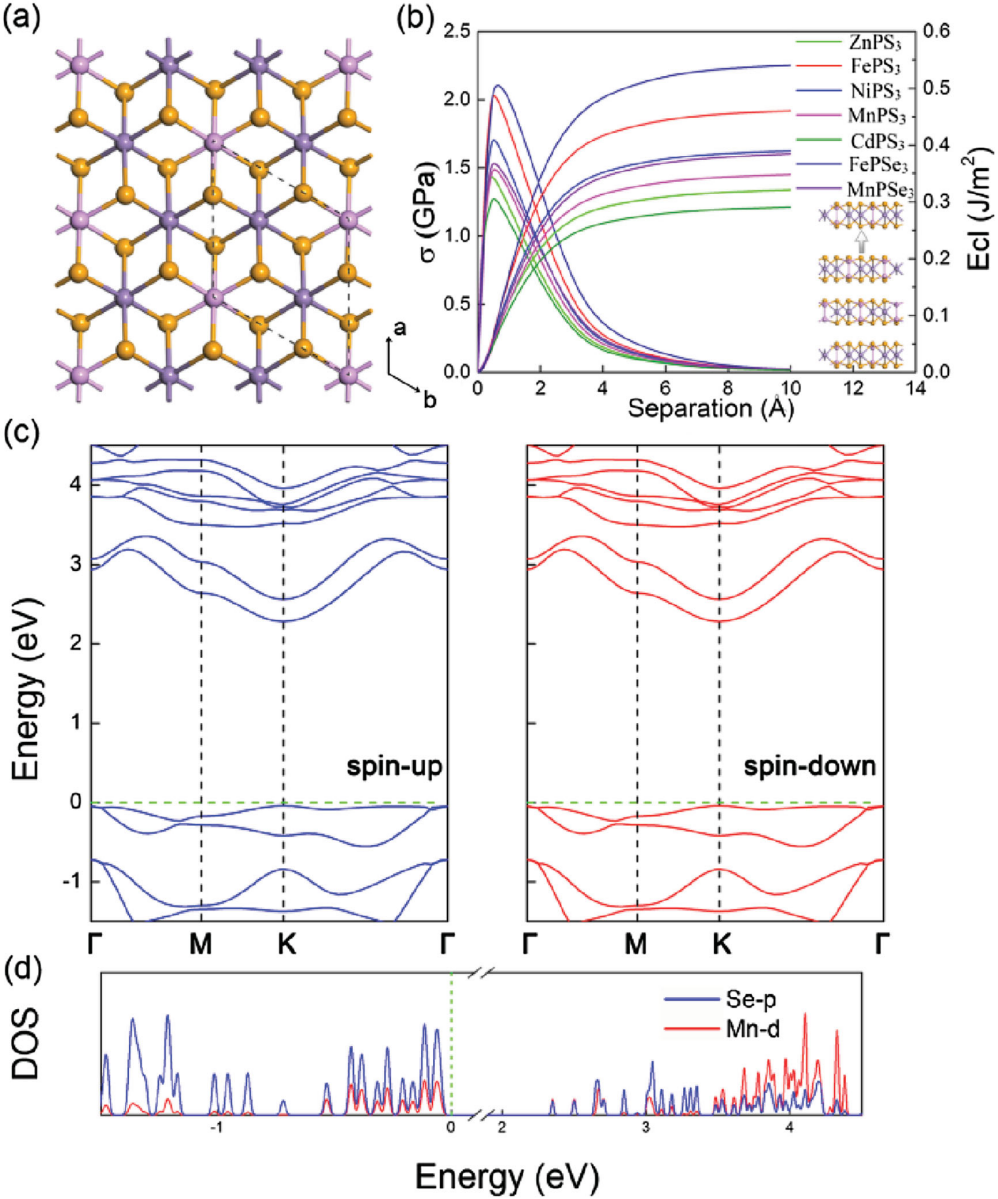
a) Monolayer structure of MPS_3_ (M = Fe, Mn, Ni, Cd, Zn) and MPSe_3_ (M = Fe, Mn). The pink, orange, and purple balls represent the P, Se/S, and M atoms, respectively. The area circulated by dashes represents the hexagonal primitive cell; b) Cleavage energy *E*_cl_ (right longitudinal coordinates) and its derivative σ (left longitudinal coordinates) as a function of the separation distance in MPS_3_ and MPSe_3_ bulk. Inset: geometry of introduced fracture; c) band structures and d) partial density of states (PDOS) near Fermi level for MnPSe_3_ monolayer.

To check the stability of the monolayers, the phonon spectra along the high‐symmetry points in Brillouin zone were computed, and are shown in Figure S1 (Supporting Information). There are no imaginary phonon modes in the phonon spectra of MPS_3_ (M = Fe, Mn, Ni, Cd, Zn) and MPSe_3_ (M = Fe, Mn) monolayers, which reveals that these monolayers are dynamically stable configurations and could be realized in experiments.

Another important issue to confirm the stability of 2D materials is whether it can form a freestanding monolayer. Therefore, the in‐plane stiffness should be high enough to withstand its own weight or even external load without substrates. To check this, the in‐plane stiffness was calculated by $C_{\mathrm{2D}}\;=\;(\partial^{2}E_{\mathrm{total}}/\partial \varepsilon^{2}){\mathrm{/S}}_{0}$, where *E*_total_, *ε*, and *S*_0_ represent the total energy per unit cell, uniaxial strain, and the area of the optimized unit cell, respectively. The calculated in‐plane stiffness is shown in Figure S2 and Table S1 of the Supporting Information. The in‐plane stiffness of sulfides is higher than that of the corresponding selenides, which might result from the greater electronegativity of sulfur. Through the elasticity theory, an estimate for the typical out‐of‐plane deformation *h*/*l* ≈ (*ρgl*/*C*_2D_)^1/3^,^18^ where *l* is the length of the monolayer, *ρ* is the density, and *g* is the gravitational acceleration. Assuming the length of the monolayer *l* to be 100 μm, the ratio of the vertical deformation and the length *h*/*l* is shown in Table S1 of the Supporting Information, which indicates that MPS_3_ (M = Fe, Mn, Ni, Cd, Zn) and MPSe_3_ (M = Fe, Mn) monolayers have sufficient rigidity to form freestanding 2D monolayers without substrates.

Then the possibility of mechanical exfoliation from the bulk was checked by calculating the cleavage energy. The relative energy of the bulk increases with the separation distance and converges to the ideal cleavage energy gradually. The optimized lattice constants for the bulk structures are shown in Table S2 of the Supporting Information, which are consistent with the experimental data indicating the credibility of the calculations. As shown in Figure 1b, the cleavage energy (0.29–0.54 J m^−2^) is comparable to the experimentally estimated value of graphite (≈0.37 J m^−2^),^19^ meaning that it is not very difficult to exfoliate monolayers from the bulk in experiments. The theoretical cleavage strength σ could be obtained by computing the maximum derivative of *E*_cl_ as shown in Figure 1b. The cleavage strength of MPS_3_ (M = Fe, Mn, Ni, Cd, Zn) and MPSe_3_ (M = Fe, Mn) (1.2–2.1 GPa) is even lower than that of graphite (≈2.1 GPa),^20^ which further indicates that the exfoliation of bulk is feasible in experiments.

We next investigated the electronic properties of the monolayers. The band structures of MPS_3_ (M = Fe, Mn, Ni, Cd, Zn) and MPSe_3_ (M = Fe, Mn) monolayers computed with Heyd–Scuseria–Ernzerhof (HSE06) functional are shown in Figure 1c and **Figure** **2**.

**Figure 2:**
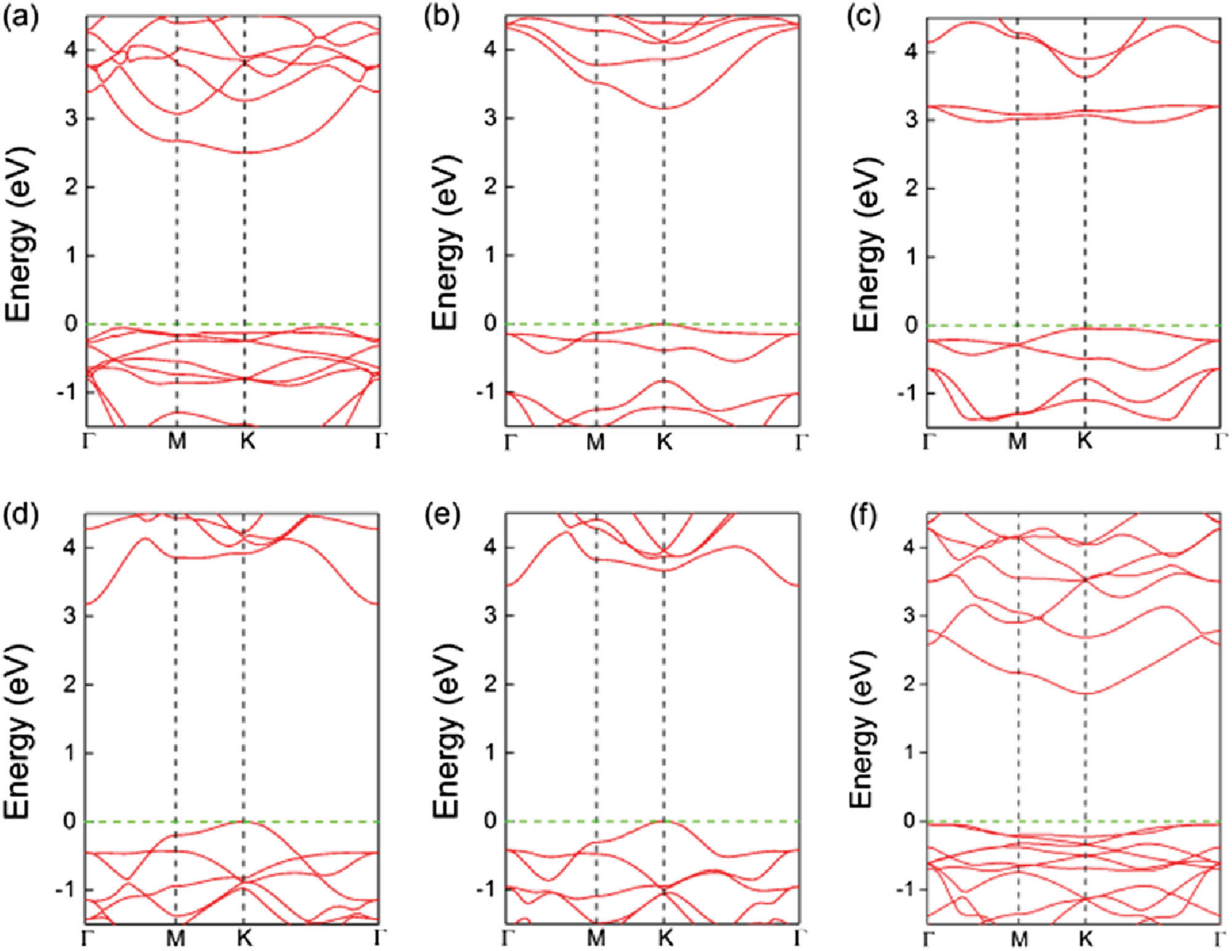
Band structures near Fermi level for a) FePS_3_, b) MnPS_3_, c) NiPS_3_, d) CdPS_3_, e) ZnPS_3_, and f) FePSe_3_ monolayers.

As shown in Table S3 of the Supporting Information, MnPS_3_ and MnPSe_3_ monolayers are direct‐band‐gap semiconductors with the band gap of 3.14 and 2.32 eV, respectively, consistent with the previous report.^12^ The valence band maximum (VBM) and the conduction band minimum (CBM) are both located at the K point. Other monolayers are indirect‐band‐gap semiconductors. More details are provided in the Supporting Information.

To further understand the compositions of VBM and CBM for MPS_3_ and MPSe_3_ mono­layers, the partial density of states (PDOS) are computed. As shown in Figure 1d and Figure S3 of the Supporting Information, the CBM and VBM of all the monolayers mainly originate from the *p* states of S/Se and the *d* states of metal atoms, respectively.

The band structures and PDOS indicate that the band gaps of MPS_3_ (M = Fe, Mn, Ni, Cd, Zn) and MPSe_3_ (M = Fe, Mn) monolayers range from 1.90 to 3.44 eV, which exceed the free energy of water splitting of 1.23 eV. In addition to the magnitude of the band gap, the band edges must straddle the redox potentials of water. To check this, the work functions of these monolayers were calculated and are shown in Table S4 of the Supporting Information.

For the water splitting reaction, the redox potential depends on the pH value.[[qv: 16b]],^21^ The standard reduction potential for H^+^/H_2_ was calculated by $E_{H^{+}{\mathrm{/H}}_{2}}^{\mathrm{red}}=\;-4.\mathrm{44}\;\mathrm{eV}\;+\;\mathrm{pH}\;\times \;0.\mathrm{059}\;\mathrm{eV}$ and the oxidation potential for O_2_/H_2_O was calculated by $E_{O_{2}{\mathrm{/H}}_{2}O}^{\mathrm{ox}}\;=\;-5.\mathrm{67}\;\mathrm{eV}\;+\;\mathrm{pH}\;\times \;0.\mathrm{059}\;\mathrm{eV}$. Considering that sulfides and selenides might be unstable in an acidic environment, the redox potential for water splitting reaction at neutral environment (pH = 7) was also calculated. The schematic diagram is shown in **Figure** **3** for the positions of band edges of MPS_3_ (M = Fe, Mn, Ni, Cd, Zn) and MPSe_3_ (M = Fe, Mn) monolayers for photocatalytic water splitting. Except FePSe_3_, whose CBM is lower than the reduction potential of H^+^/H_2_ at pH = 7, the band edges of the rest compounds straddle the redox potentials of water at pH = 0 and 7. The results indicate that these materials are candidates for water‐splitting photocatalysts without an external bias voltage. More fascinatingly, besides the advantages of suitable positions of band edges at both acidic and neutral environment, MnPS_3_ and MnPSe_3_ are direct‐band‐gap semiconductors.

**Figure 3:**
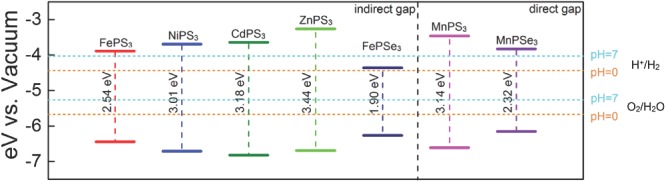
The location of VBM and CBM calculated with HSE06 functional of MPS_3_ and MPSe_3_ monolayers. The redox potentials of water splitting at pH = 0 (orange dashed lines) and pH = 7 (cyan dashed lines) are shown for comparison.

Another very important condition for photocatalytic water splitting is that the materials should capture a significant fraction of visible spectrum because it counts for more than 40% of the solar spectrum.

To investigate the performance under light, the optical absorption coefficient was calculated and is shown in **Figure** **4**. The corresponding imaginary parts *ε*_2_ of the dielectric function is shown in Figure S4 of the Supporting Information. The absorption coefficient is defined as the decay of light intensity spreading in a unit length of medium. FePSe_3_, MnPSe_3_, FePS_3_, and NiPS_3_ monolayers exhibit obvious optical absorption in visible spectrum and among them, the absorption of FePSe_3_ and MnPSe_3_ is stronger. However, FePSe_3_ might have no photocatalytic activity for water splitting at neutral environment. For MnPSe_3_, there are two absorption peaks in visible spectrum, indicating the strong optical absorption. Besides, the direct gap would make MnPSe_3_ more advantageous over other materials. The results indicate that MnPSe_3_ monolayer would exhibit better performance for photocatalytic water splitting. To investigate the effects of the layer number on the electronic properties and optical absorption of MnPSe_3_, MnPSe_3_ bilayer was considered. More details are shown in Figures S5–S7 of the Supporting Information. The results indicate that MnPSe_3_ bilayer could also exhibit catalytic activity for photocatalytic water splitting under visible light.

**Figure 4:**
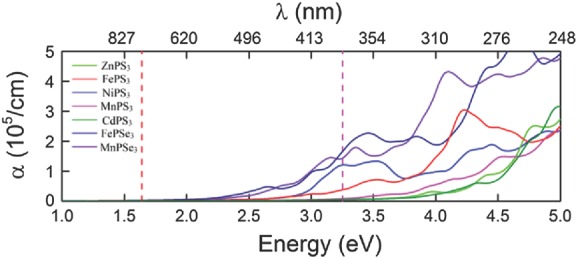
Optical absorption coefficient α for MPS_3_ (M = Fe, Mn, Ni, Cd, Zn) and MPSe_3_ (M = Fe, Mn). The area between the red and the purple lines represents the visible range.

To further evaluate the performance of MnPSe_3_ monolayer as photocatalysts, the carrier effective mass and carrier mobility were investigated for quantitative evaluation on the ability to transfer electron/hole along the specific direction.

The effective masses of electrons (*m*_e_*) and holes (*m*_h_*) are calculated by fitting parabolic functions to CBM and VBM, respectively. As shown in **Table** **1**, *m*_e_* and *m*_h_* along the direction of a (Figure 1a) were calculated, which are in general smaller than those of some common photocatalysts.^22^ The smaller effective mass indicates that the transfer of carriers to the reactive sites would be easier in the photocatalytic process.

**Table 1:** Effective mass |m*| (*m*_e_, the mass of free electrons), in‐plane stiffness *C*_2D_ (N m^−1^), DP constant |*E*_1_| (eV), and carrier mobility *μ* (cm^2^ V^−1^ S^−1^) for electrons and holes along the direction of a (Figure 1a)

	|m*|	*C*_2D_	|*E*_1_|	*μ*
Electrons (K→Γ)	0.55	60.8	2.12	625.9
Holes (K→Γ)	1.22	60.8	4.08	34.7

To compute the DP constant *E*_1_, the VBM and CBM position of MnPSe_3_ monolayer with respect to the vacuum level as a function of the uniaxial strain *ε* along a direction is shown in Figure S8 of the Supporting Information. On the basis of the obtained |m*|, *C*_2D_, and |*E*_1_|, the calculated carrier mobility of the 2D MnPSe_3_ monolayer at room temperature (*T* = 300 K) is shown in Table 1. The electron mobility is 625.9 cm^2^ V^−1^ S^−1^ while the hole mobility is 34.7 cm^2^ V^−1^ S^−1^. The carrier mobility of MnPSe_3_ monolayer could be comparable to or even higher than that of many other 2D semiconductors, such as MoS_2_ monolayer (≈200 cm^2^ V^−1^ S^−1^),^23^ hydrogenated graphene (≈105 cm^2^ V^−1^ S^−1^), fluorinated graphene (≈45 cm^2^ V^−1^ S^−1^), BN (≈487 cm^2^ V^−1^ S^−1^), and BC_2_N (≈180 cm^2^ V^−1^ S^−1^).^24^ Moreover, the huge difference between the carrier mobility of electrons and holes indicates that the effective separation of electron–hole pairs and the small probability of recombination for photogenerated carriers. In addition, 2D materials are very promising candidates as photocatalysts, with high specific surface area available for photocatalytic reactions, short distance for the generated electrons and holes to migrate, and further reduced probability in the recombination of photogenerated carriers.[[qv: 15b]]

Overall, MnPSe_3_ monolayer has many features, strong absorption in visible‐light spectrum, photocatalytic water splitting into H_2_ and O_2_ simultaneously, broad applicability (pH = 0–7), high carrier mobility, and feasible synthesis in experiments. Thus, MnPSe_3_ monolayer is a promising candidate as photocatalysts for water splitting.

In conclusion, the structure and stability of 2D metal phosphorus trichalcogenides monolayers were explored by first‐principles computations, which indicate that these 2D monolayers could be obtained in experiments by exfoliating the corresponding bulk. The calculated band gaps and band edge positions from accurate HSE06 functional predict that MPS_3_ (M = Fe, Mn, Ni, Cd, Zn) and MPSe_3_ (M = Fe, Mn) mono­layers are promising candidates as photocatalysts for water splitting. Particularly, MnPSe_3_ monolayer is a direct‐band‐gap semiconductor which exhibits obvious absorption in visible‐light spectrum. Moreover, our calculations of effective mass and carrier mobility for MnPSe_3_ monolayer illustrate the transfer of carriers to the reactive sites would be easier and the probability of recombination would be lower for photogenerated carriers in the photocatalytic process. These results reflect that 2D MnPSe_3_ monolayer could be a promising photocatalyst for water splitting.

## Experimental Section

Our first‐principles computations based on density functional theory (DFT) were performed with a plan‐wave basis set as implemented in the Vienna ab initio simulation package (VASP).^25^ The projector augmented wave (PAW) was used to describe the ion–electron interaction.^26^ The generalized gradient approximation (GGA) expressed by the functional of Perdew, Burke, and Ernzerhof (PBE).^27^ A 500 eV cutoff was used for the plane‐wave basis set. The DFT‐D3 method with Becke–Jonson damping was adopted to accurately account for the van der Waals force for weak interactions.^28^ A Monkhorst–Pack k‐point mesh of 7 × 7 × 1 was used for 2D sheet, 7 × 7 × 2 for selenide bulk and 6 × 3 × 5 for sulfide bulk. To study 2D systems under periodic boundary conditions (PBC), a vacuum space with at least 15 Å was inserted between the MPX_3_ sheets and the periodically repeated images. Moreover, considering that GGA functional systematically underestimates the band gaps,^29^ we computed the band structures with the HSE06 hybrid functional.^30^ The computation of the phonon dispersion spectrums was calculated through CASTEP code with finite displacement method as implemented in Materials Studio.^31^

To investigate the optical absorption, the imaginary part of dielectric function *ε*_2_ was calculated. The expression for *ε*_2_ was given as (1)
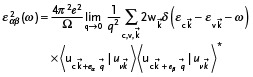
where the indices c and v refer to the conduction and valence band states, respectively, which were determined by the HSE06 functional here, and $u_{\mathrm{ck}}^{\to }$is the cell periodic part of the orbital at the k‐point ${}_{k}^{\to }$. The absorption coefficient α(ω) was calculated by^32^

(2) $$\alpha \left(\omega \right)\;=\;\sqrt{2}\omega {\left(\sqrt{\varepsilon_{1}^{2}\left(\omega \right)\;+\;\varepsilon_{2}^{2}\left(\omega \right)}-\varepsilon_{1}\left(\omega \right)\right)}^{\mathrm{1/2}}$$

where *ε*_1_ is the real part of dielectric function, which could be obtained from *ε*_2_ by Kramer–Kronig relationship.

For inorganic semiconductors, the electron coherence length is close to the acoustic phonon wavelength, which is much longer than the bonds. As a result, phonon scattering dominates the intrinsic mobility which can be described by the deformation potential theory.^33^ For 2D materials, the carrier mobility is given by (3)
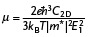
where *T* is the temperature, 300 K was adopted in this study, *e* is the electron charge, and *h–* is the reduced Planck constant. m* is the effect mass defined as m* = *h–* ^2^(∂^2^ *E*(*k*)/∂*k* ^2^)^−1^, where *k* is the wave vector, and *E*(*k*) is the energy corresponding to the wave vector *k*. *E*_1_ is the DP constant denoting the shift of band edges induced by uniaxial strain, *E*_1_ = ∂*E*_edge_/∂*ε*. *C*_2D_ is the in‐plane stiffness as shown in Table S1 of the Supporting Information.

## Supporting information

As a service to our authors and readers, this journal provides supporting information supplied by the authors. Such materials are peer reviewed and may be re‐organized for online delivery, but are not copy‐edited or typeset. Technical support issues arising from supporting information (other than missing files) should be addressed to the authors.

Supplementary
