# Supplementary material for: MnPSe3 Monolayer: A Promising 2D Visible‐Light Photohydrolytic Catalyst with High Carrier Mobility
Source: Adv Sci (Weinh). 2016 Apr 23;3(10):1600062. doi: 10.1002/advs.201600062 (PMC5096250; doi:10.1002/advs.201600062)
Supplement: Supplementary file 1 — Supplementary [file ADVS-3-0o-s001.pdf]

Copyright WILEY-VCH Verlag GmbH & Co. KGaA, 69469 Weinheim, Germany, 2016.

Supporting Information

for *Adv. Sci.,* DOI: 10.1002/advs.201600062

$MnPSe_3$ Monolayer: A Promising 2D Visible-Light Photohydrolytic Catalyst with High Carrier Mobility

*Xu Zhang, Xudong Zhao, Dihua Wu, Yu Jing, and Zhen Zhou**

Supporting Information

MnPSe3 Monolayer: A Promising 2D Visible-Light Photo-hydrolytic Catalyst with High Carrier Mobility

Xu Zhang, Xudong Zhao, Dihua Wu, Yu Jing and Zhen Zhou*

Tianjin Key Laboratory of Metal and Molecule Based Material Chemistry, Computational Centre for Molecular Science, Institute of New Energy Material Chemistry, Collaborative Innovation Center of Chemical Science and Engineering (Tianjin), School of Materials Science and Engineering, National Institute for Advanced Materials, Nankai University, Tianjin 300350, P.R. China

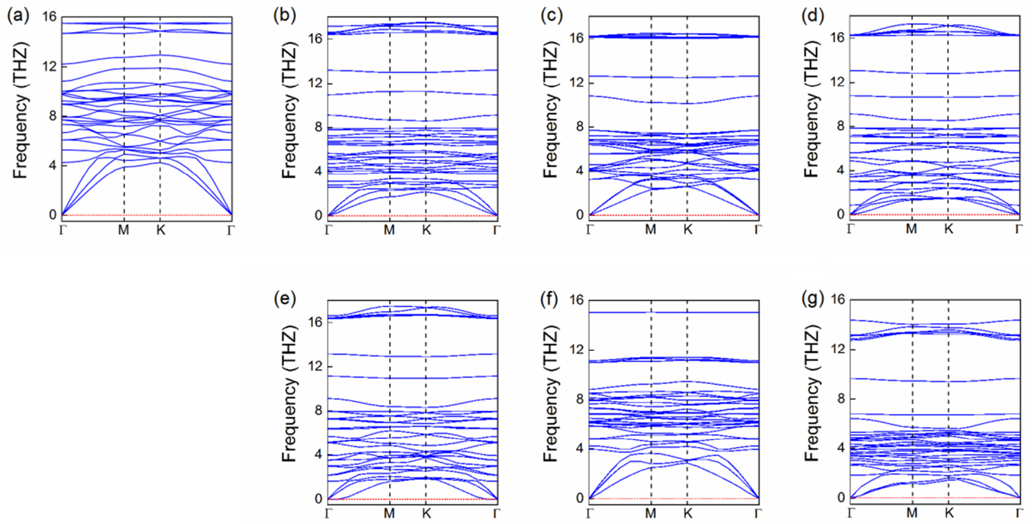

Figure S1. Phonon dispersion spectrum of (a) $FePS_3$; (b) $MnPS_3$; (c) $NiPS_3$; (d) $CdPS_3$; (e) $ZnPS_3$; (f) $FePSe_3$ and (g) $MnPSe_3$ monolayer. The fractional coordinates of high-symmetry points: Γ (0, 0, 0), M (0, 0.5, 0) and K (-1/3, 2/3, 0).

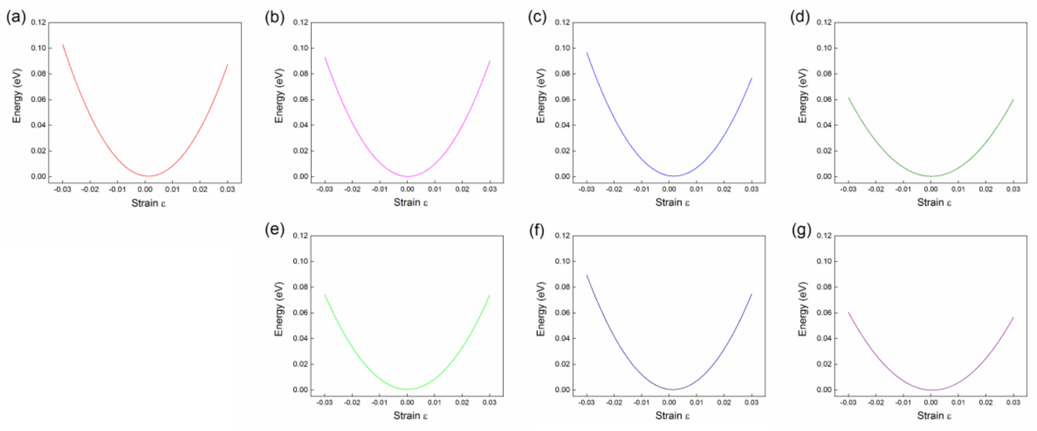

Figure S2. The relationship between energy and uniaxial strain for (a) $FePS_3$; (b) $MnPS_3$; (c) $NiPS_3$; (d) $CdPS_3$; (e) $ZnPS_3$; (f) $FePSe_3$ and (g) $MnPSe_3$ monolayer. The solid line is a quadratic fitting to the data.

Table S1. The in-plane stiffness C_{2D} (N/m), density ρ (10^{-6} kg/m^2) and h/l (10^{-4}) of MPS_3 and $MPSe_3$.

	$FePS_3$	$MnPS_3$	$NiPS_3$	$CdPS_3$	$ZnPS_3$	$FePSe_3$	$MnPSe_3$
C_{2D}	119.7	107.7	106.8	64.4	85.6	90.2	60.8
ρ	2.16	2.00	2.15	2.38	2.09	3.34	3.12
h/l	2.61	2.63	2.70	3.31	2.88	3.31	3.69

Table S2. The lattice parameters a, b, c (Å) and space group of bulk MPS_3 and $MPSe_3$. The numbers in brackets are experimental data.[1]

	a	b	c	space group
$FePS_3$	5.72 (5.95)	9.90 (10.30)	6.55 (6.72)	C2/m
$MnPS_3$	5.93 (6.08)	10.36 (10.52)	6.62 (6.80)	C2/m
$NiPS_3$	5.76 (5.81)	9.97 (10.07)	6.63 (6.42)	C2/m
$CdPS_3$	6.21 (6.22)	10.75 (10.76)	6.85 (6.87)	C2/m
$ZnPS_3$	5.93 (5.97)	10.26 (10.34)	6.73 (6.76)	C2/m
$FePSe_3$	6.18 (6.27)	6.18 (6.27)	19.75 (19.81)	R $\bar{3}$
$MnPSe_3$	6.29 (6.39)	6.29 (6.39)	19.77 (19.99)	R $\bar{3}$

Table S3. The band gaps G (eV), position of VBM and CBM for MPS_3 and $MPSe_3$.

	$FePS_3$	$MnPS_3$	$NiPS_3$	$CdPS_3$	$ZnPS_3$	$FePSe_3$	$MnPSe_3$
G	2.54	3.14	3.01	3.18	3.44	1.90	2.32
VBM	K→Γ	K	K	K	K	Γ	K
CBM	K	K	K→Γ	Γ	Γ	K	K
	indirect	direct	indirect	indirect	indirect	indirect	direct

For $CdPS_3$ and $ZnPS_3$ monolayer, the VBM is located at the K point while the CBM is located at the Γ point, indicating an indirect band gap. Conversely, the VBM is located at the Γ point and the CBM is located at the K point for $FePSe_3$ monolayer. $FePS_3$ monolayer is an indirect semiconductor with a band gap of 2.54 eV with the CBM is located at K point and the VBM on the K→Γ path. Lastly, $NiPS_3$ has an indirect band gap of 3.01 eV and the VBM is located at the K point while the CBM lies on the K→Γ path.

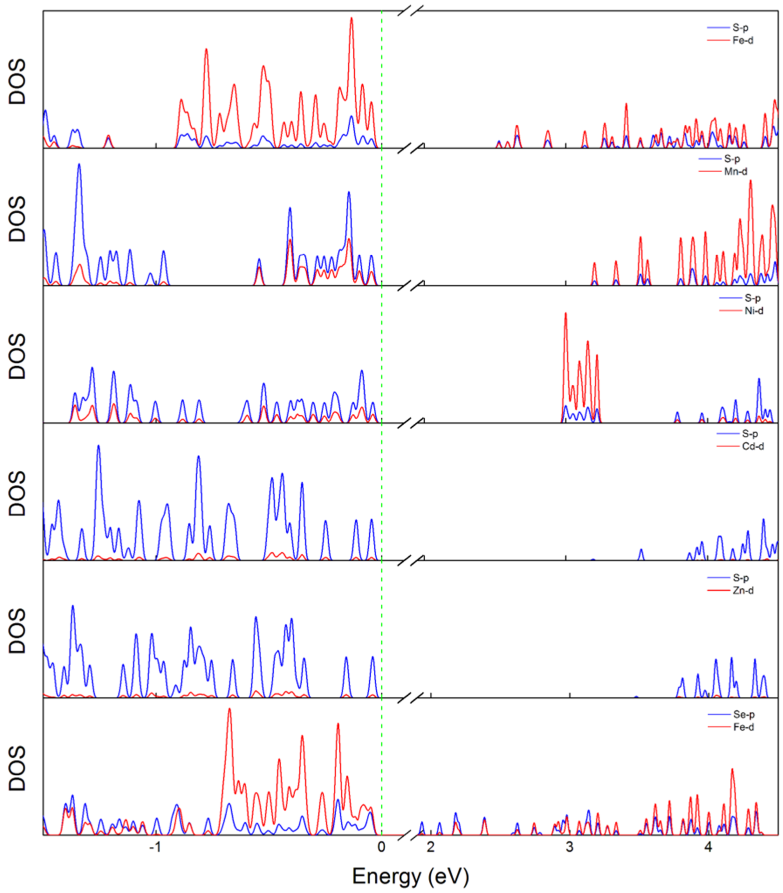

Figure S3. The partial density of states of (a) $FePS_3$; (b) $MnPS_3$; (c) $NiPS_3$; (d) $CdPS_3$; (e) $ZnPS_3$ and (f) $FePSe_3$ monolayer.

For the VBM of $FePS_3$ and $FePSe_3$ monolayers, the d-states of Fe contribute more. For the VBM of other monolayers, the p-states of S/Se contribute more. For the CBM of $MnPS_3$, $MnPSe_3$ and $NiPS_3$ monolayers, the d-states of metal atoms contribute more and for the CBM of other monolayers, the p-states of S/Se contribute more.

Table S4. The work functions (eV) of MPS_3 and $MPSe_3$ and the band edges (eV) with respect to the vacuum level calculated by HSE06 functional.

	$FePS_3$	$MnPS_3$	$NiPS_3$	$CdPS_3$	$ZnPS_3$	$FePSe_3$	$MnPSe_3$
work function	6.40	6.60	6.16	6.81	6.69	5.16	6.11
CBM	-3.89	-3.46	- 3.69	-3.64	-3.26	-4.36	-3.83
VBM	-6.44	-6.61	-6.71	-6.82	-6.69	-6.26	-6.15

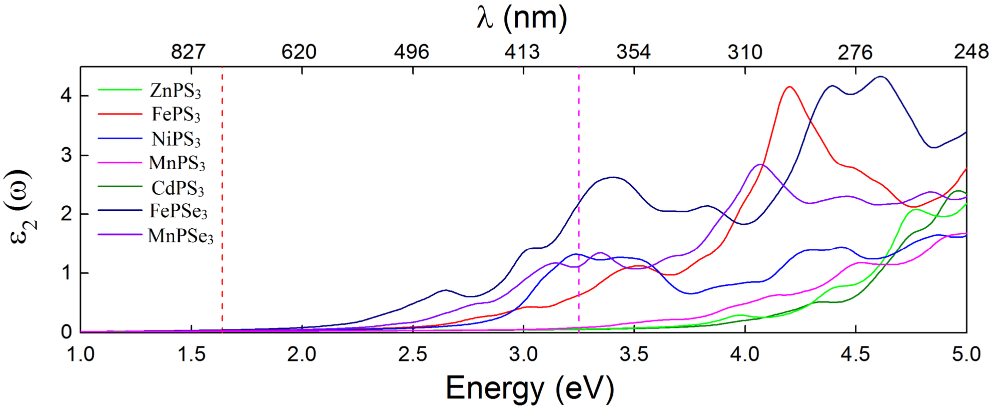

Figure S4. Imaginary parts ε_2 of the dielectric function for MPS_3 (M = Fe, Mn, Ni, Cd, Zn) and $MPSe_3$ (M = Fe, Mn).The area between the red and the purple lines represents the visible range.

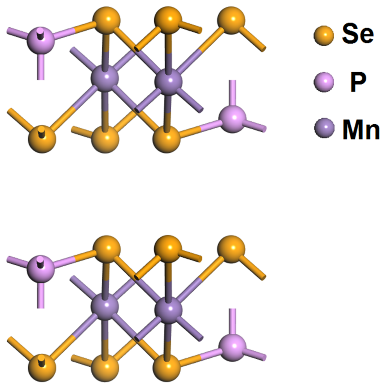

Figure S5. Side view of optimized geometries of $MnPSe_3$ bilayer.

Several kinds of structures were considered to find the most stable configuration. As shown in Figure S5, the configuration that two layers are arranged in the same way is the most stable among the considered structures.

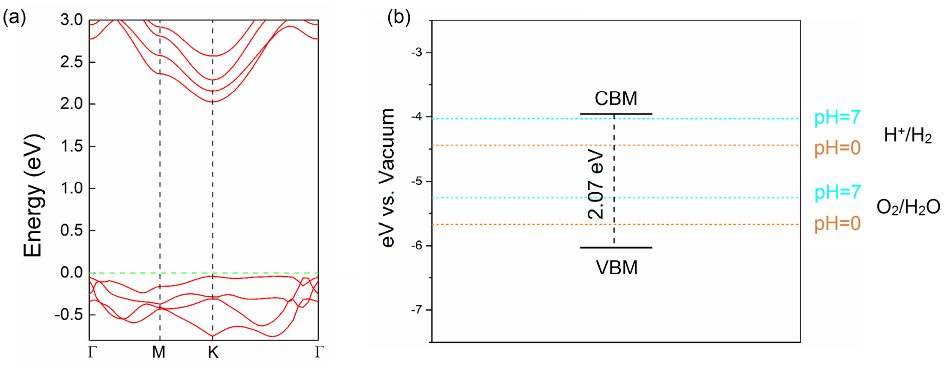

Figure S6. (a) Band structure and (b) the location of VBM and CBM calculated with HSE06 functional of $MnPSe_3$ bilayer. The redox potentials of water splitting at pH = 0 (orange dashed lines) and pH = 7 (cyan dashed lines) are shown for comparison.

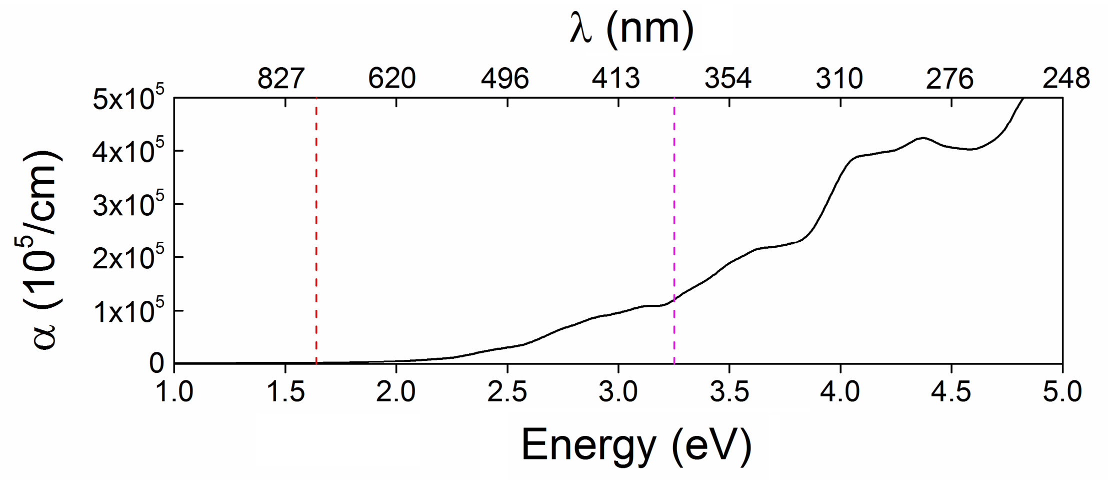

Figure S7. Optical absorption coefficient α for $MnPSe_3$ bilayer. The area between the red and the purple lines represents the visible range.

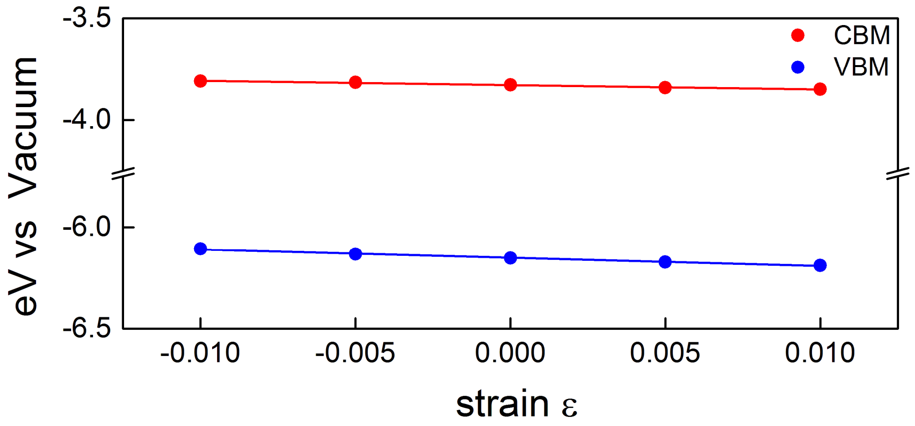

Figure S8. The CBM and VBM position of $MnPSe_3$ monolayer with respect to the vacuum level as a function of the uniaxial strain ε along a direction. The solid lines are linear fitting to the data.

[1] a) A. Wiedenmann, J. Rossat-Mignod, A. Louisy, R. Brec, J. Rouxel, *Solid State Commun.* **1981**, *40*, 1067-1072; b) G. Ouvrard, R. Brec, J. Rouxel, *Mater. Res. Bull.* **1985**, *20*, 1181-1189; c) F. Boucher, M. Evain, R. Brec, *J. Alloys Compd.* **1994**, *215*, 63-70.
